# Supplementary material for: Measuring the well-being of health care professionals in the Punjab: a psychometric evaluation of the Warwick–Edinburgh Mental Well-being Scale in a Pakistani population
Source: PeerJ. 2015 Oct 1;3:e1264. doi: 10.7717/peerj.1264 (PMC4636413; doi:10.7717/peerj.1264)
Supplement: Table S1 [file peerj-03-1264-s001.docx]

| Table S1. Factor loadings for the 14 items in the Warwick-Edinburgh Mental Well-being Scale in a sample of Pakistani health care providers (n=1271) | | |
| --- | --- | --- |
|  | Factor | |
|  | 1 | 2 |
| I’ve been feeling optimistic about the future | .509 |  |
| I’ve been feeling useful | .581 |  |
| I’ve been feeling relaxed | .557 | .333 |
| I’ve been feeling interested in other people | .467 | .424 |
| I’ve had energy to spare | .451 | .382 |
| I’ve been dealing with problems well | .661 |  |
| I’ve been thinking clearly | .694 |  |
| I’ve been feeling good about myself | .743 |  |
| I’ve been feeling close to other people | .650 |  |
| I’ve been feeling confident | .733 |  |
| I’ve been able to make up my own mind about things | .651 |  |
| I’ve been feeling loved | .582 |  |
| I’ve been interested in new things | .573 |  |
| I’ve been feeling cheerful | .687 |  |
| Extraction Method: Principal Axis Factoring.  Rotation Method: Quartimax with Kaiser Normalization. | | |
| Rotation converged in 3 iterations. | | |
